# 3D bioprinting of mineralizing cyanobacteria as novel approach for the fabrication of living building materials

**DOI:** 10.3389/fbioe.2023.1145177

**Published:** 2023-04-03

**Authors:** Olena Reinhardt, Stephanie Ihmann, Matthias Ahlhelm, Michael Gelinsky

**Affiliations:** ^1^ Centre for Translational Bone, Joint and Soft Tissue Research, Faculty of Medicine Carl Gustav Carus, Technische Universität Dresden, Dresden, Germany; ^2^ Biologized Materials and Structures, Fraunhofer Institute for Ceramic Technologies and Systems IKTS, Dresden, Germany

**Keywords:** biomineralization, three-dimensional printing, living building materials, engineered living materials, biocement, *Synechococcus* sp, MICP, microbially induced carbonate precipitation

## Abstract

Living building materials (LBM) are gaining interest in the field of sustainable alternative construction materials to reduce the significant impact of the construction industry on global CO_2_ emissions. This study investigated the process of three-dimensional bioprinting to create LBM incorporating the cyanobacterium *Synechococcus* sp. strain PCC 7002, which is capable of producing calcium carbonate (CaCO_3_) as a biocement. Rheology and printability of biomaterial inks based on alginate-methylcellulose hydrogels containing up to 50 wt% sea sand were examined. PCC 7002 was incorporated into the bioinks and cell viability and growth was characterized by fluorescence microscopy and chlorophyll extraction after the printing process. Biomineralization was induced in liquid culture and in the bioprinted LBM and observed by scanning electron microscopy, energy-dispersive X-ray spectroscopy, and through mechanical characterization. Cell viability in the bioprinted scaffolds was confirmed over 14 days of cultivation, demonstrating that the cells were able to withstand shear stress and pressure during the extrusion process and remain viable in the immobilized state. CaCO_3_ mineralization of PCC 7002 was observed in both liquid culture and bioprinted LBM. In comparison to cell-free scaffolds, LBM containing live cyanobacteria had a higher compressive strength. Therefore, bioprinted LBM containing photosynthetically active, mineralizing microorganisms could be proved to be beneficial for designing environmentally friendly construction materials.

## 1 Introduction

Using a variety of materials and a variety of structures and geometries created from 3D model data, the additive manufacturing (AM) process of three-dimensional (3D) printing opens up a vast range of potential applications (Murphy and Atala, 2014; Ngo et al., 2018; Praveena et al., 2022). The construction industry has recently developed interest in the AM technology (Khoshnevis, 2004; Wu et al., 2016; Kazemian et al., 2017). The benefits include freedom of design, individuality, and complexity of the structures (Labeaga-Martínez et al., 2017; Ngo et al., 2018). Concrete is the most common material utilized in building with AM (Schutter et al., 2018). It is easy to process when it is fresh and reaches high compressive strength when cured (Guamán-Rivera et al., 2022). However, the building sector is responsible for 39% of global carbon dioxide (CO_2_) emissions, with concrete alone accounting for 11% of global CO_2_ emissions and thus significantly contributing to climate change (Concrete needs to lose its colossal carbon footprint, 2021; Architecture, 2022). Due to population growth and progressing urbanization, global demand for concrete will increase by up to 23% by 2050 (Architecture, 2022). To limit further global warming to +2°C by 2050, a significant reduction of CO_2_ emissions by 24% is required. Therefore, new innovative technologies are needed that enable the reuse of existing materials (Tam, 2008; Noguchi et al., 2011), the reduction of total material demand (Khan et al., 2012; Panesar and Zhang, 2020; Zeybek et al., 2022), and the use of carbon-depleting materials (IEA, 2018; Concrete needs to lose its colossal carbon footprint, 2021).

**FIGURE 1:**
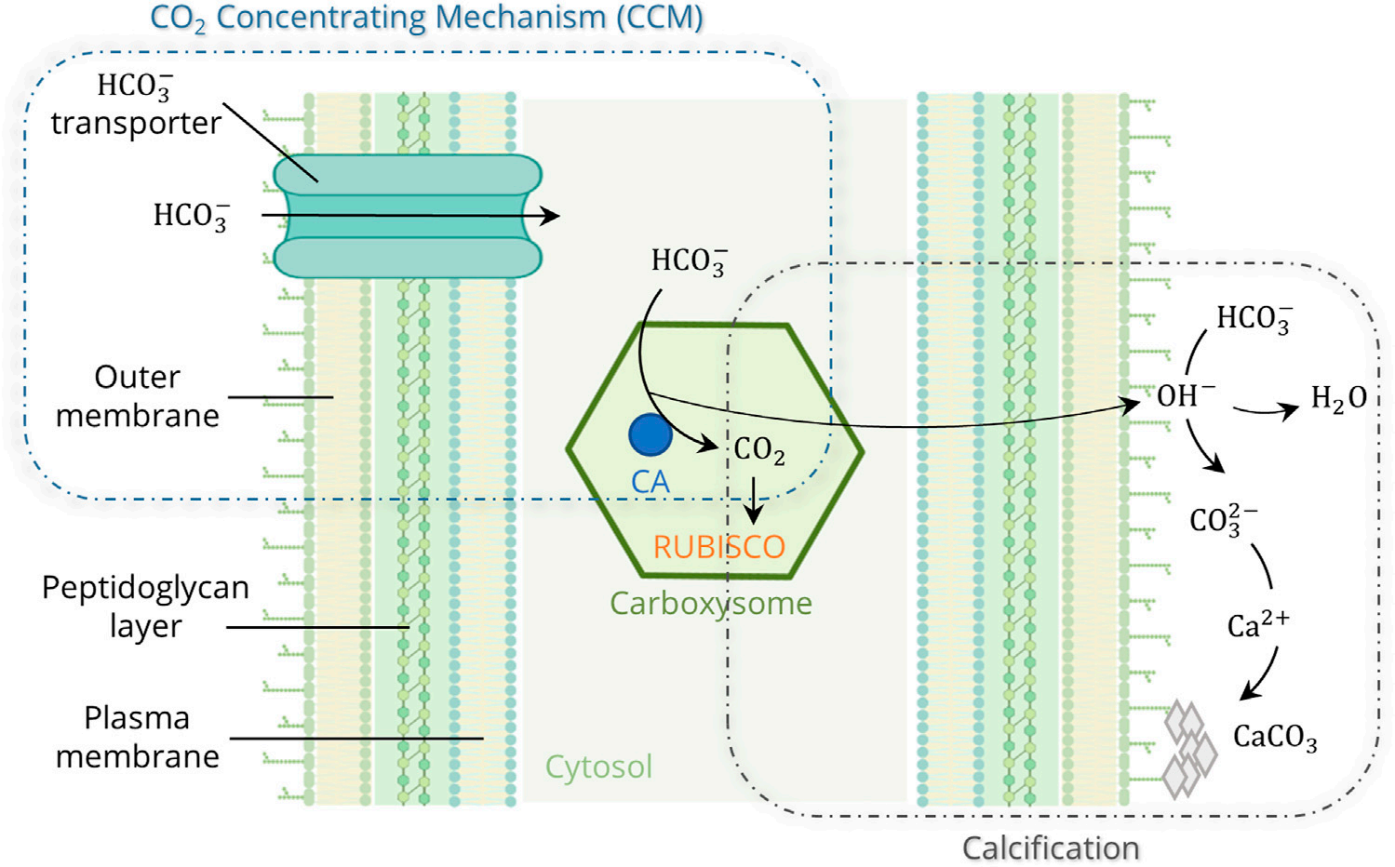
Model of the cyanobacterial calcification, enhanced by photosynthetic CO_2_ Concentrating Mechanisms (CCM) (created in BioRender.com; modified from Riding, 2006 and Kamennaya et al., 2012).

A look at nature shows that worldwide there are natural producers of a biological cement consisting mainly of calcium carbonate (CaCO_3_) (Strong and Eadie, 1978; Thompson et al., 1997; Foster et al., 2009). One of the oldest types of mineralizing microorganisms on Earth are cyanobacteria, which have been detected in about 2.8 billion years old fossil mats called stromatolites. Because they generate energy through oxygenic photosynthesis, enormous amounts of oxygen were produced as a waste product of their metabolism, allowing the first aerobic life forms to emerge. Cyanobacteria are thus considered to have paved the way for life (Tetsch, 2016). Even today, they are of great ecological importance, sequestering about 40% of annual CO_2_ emissions from the atmosphere (Falkowski, 1994). Over 25% of global photosynthesis is carried out by only two genera, *Prochlorococcus* and *Synechococcus* (Whitman et al., 1998; Partensky et al., 1999). In addition to photosynthesis, the second pathway in cyanobacteria for CO_2_ fixation is Microbially Induced Carbonate Precipitation (MICP) (Golubic, 1973; Pentecost and Riding, 1986; Merz, 1992). This natural calcification occurs, for example, in stromatolites or whiting events, where cyanobacteria grow exponentially and mineralize dissolved inorganic carbon (HCO_3_^−^ or CO_3_^2-^) to calcium carbonate (CaCO_3_) in the presence of high Ca^2+^ concentrations (Robbins and Blackwelder, 1992; Thompson et al., 1997; Foster et al., 2009). Cyanobacteria reduce the kinetic barriers and promote mineral formation through various metabolic reactions. They can cope with low CO_2_ concentrations due to carbon concentrating mechanisms (CCM) that actively transport inorganic carbon compounds (HCO_3_^−^) into the cell, where they accumulate in the carboxysomes and are converted to CO_2_ by the enzyme Carbonic Anhydrase (CA) (Figure 1). CCM increase the local CO_2_ concentration at the carboxylation site of the enzyme ribulose-1,5-biphosphate carboxylase-oxygenase (RUBISCO) by up to a thousand-fold (Badger and Price, 2003; Price et al., 2008). During photosynthesis, RUBISCO utilizes CO_2_ for energy generation (ATP). OH^−^ is released as a by-product during the conversion of HCO_3_^−^ to CO_2_, leading to an alkalization of the microenvironment. Additionally, Ca^2+^ accumulates at the cell surface, through transportation by Ca^2+^/H^+^ antiporters and chemical attraction to the negatively charged bacterial cell surface (Arp et al., 2001). These effects lead to supersaturation and the eventual formation of CaCO_3_ on the outer cell surface (Thompson and Ferris, 1990; Merz, 1992).

In addition to cyanobacteria, there are other microorganisms capable of MICP, such as sulfate-reducing bacteria (Perito and Mastromei, 2011; Alshalif et al., 2016; Tambunan et al., 2019), microorganisms that utilize organic acids (Rodriguez-Navarro et al., 2003; Chekroun et al., 2004; Zhu and Dittrich, 2016), and microorganisms that are involved in a nitrogen cycle (Ersan et al., 2015; Zhu and Dittrich, 2016). Dhami et al. (2013b) describe the ureolytic pathway as the most easily controllable mechanism with the potential for rapid CaCO_3_ production. The potential of ureolytic MICP has been analyzed in a variety of engineering applications, including sequestration of inorganic contaminants (Mitchell and Ferris, 2005), soil stabilization (DeJong et al., 2010), prevention of CO_2_ leakage from geological reservoirs (Phillips et al., 2001), and crack repair in concrete (Wang et al., 2014; Belie et al., 2018). Recently, MICP of ureolytic bacteria in 3D printed and casted structures was shown to be potentially applied as artificial corals to help restoring marine reefs (Hirsch et al., 2023). However, the major disadvantage of the ureolytic MICP is the production of ammonia as a by-product, which has potentially toxic effects on living organisms and vegetation and could lead to eutrophication or acidification of ecosystems (Dhami et al., 2013b). Another drawback is the production of endospores that can cause uncontrollable germination (Muynck et al., 2010).

Recently, photosynthetic MICP performed by cyanobacteria has received more attention in several engineering approaches because they serve as a carbon capture system by absorbing CO_2_ during growth and mineralization (Heveran et al., 2020; Beatty et al., 2022; Sidhu et al., 2022). Heveran et al. (2020) investigated the inclusion of mineralizing cyanobacteria in a gelatin-hydroge-sand mixture by gel casting and demonstrated promising results in terms of the creation of consolidated constructs, probably caused by cyanobacteria-induced CaCO_3_ mineralization, which improved the mechanical properties of such building materials. The application of MICP in the construction industry is becoming increasingly important. In the future, biomineralized cement could partly replace conventional cement in certain non-load-bearing applications to reduce overall CO_2_ emissions of building materials. Following the example of rammed earth, an ancient construction technique, where 10–15 cm of soil is deposited and rammed in layers (Nouri et al., 2021), MICP-based building materials could be created through a layer-by-layer mineralization process.

In this study, the concept of cyanobacterial MICP was combined with the manufacturing method of 3D bioprinting, to investigate the possibility of producing biological cement (CaCO_3_) in 3D printed constructs. Extrusion bioprinting with bioinks, hydrogels containing living cells, is an established technique especially in the biomedical and tissue engineering field (Malda et al., 2013; Kilian et al., 2017; Valot et al., 2019; Ahlfeld et al., 2020), but there are also increasing applications in biotechnology (Krujatz et al., 2022). Bioprinting like every AM technology allows the manufacturing of 3D constructs of predefined outer and inner morphology based on computer-aided design. In case of bioprinted tissue samples, the presence of suitable open porosity has been demonstrated to be beneficial for cell survival and development, especially in case of volumetric objects (Khalighi and Saadatmand, 2021). In this study, a bioink consisting of alginate, methylcellulose, and sea sand was used to incorporate the cyanobacterium *Synechococcus* sp. strain PCC 7002. *Synechococcus* species are ubiquitous in marine and freshwater environments, their calcification ability is well understood and has been analyzed in several studies, and they are known to be stable towards environmental stress (e.g., high irradiation: up to 4.5 mE m^-2^ s^-1^ (Nomura et al., 2006), temperature: up to 42 °C (Ludwig and Bryant, 2012), moisture (Heveran et al., 2020), and salinity changes: 3–300 mM NaCl (Ludwig and Bryant, 2012)). After the viability of the cells in the printed scaffolds was confirmed, the MICP of PCC 7002 was investigated in liquid culture and in the bioprinted structures. The calcified structures are referred to as Living Building Materials (LBM), consisting of structural materials which create the backbone and living microorganisms that transform the structural properties and add biological function (Heveran et al., 2020).

## 2 Materials and methods

### 2.1 Bacterial strain and cultivation conditions

The cyanobacterium *Synechococcus* sp. strain PCC 7002 (further referred to as PCC 7002) was purchased from the Pasteur Collection of Cyanobacteria (Institut Pasteur, Paris, France). Cell growth was maintained in A+ medium containing 0.308 M NaCl, 0.02 M MgSO_4_, 0.08 mM Na_2_EDTA, 8.05 mM KCl, 2.52 mM CaCl_2_, 11.8 mM NaNO_3_, 0.37 mM KH_2_PO_4_, 8.26 mM Trizma Base (pH 8.2) and 10 mL/L Trace Components, consisting of 55.5 mM H_3_BO_3_, 0.23 mM ZnCl_2_, 0.021 mM MoO_3_, 0.3 µM vitamin B_12_, 0.14 mM FeCl_3_, 0.22 mM MnCl_2_, 0.12 µM CuSO_4_, 0.5 µM CoCl_2_ (Stevens et al., 1973; Heveran et al., 2020). PCC 7002 was maintained in Erlenmeyer flasks in the light incubator Infors Multitron (Infors, Bottmingen, Switzerland) at 200 rpm, 24 °C, and an illumination of 180 μmol m^-2^ s^-1^ by cool white fluorescent lamps (Heveran et al., 2020).

### 2.2 Preparation and rheological characterization of the biomaterial inks

A hydrogel bioink based on alginate and methylcellulose (algMC) was prepared for extrusion-based bioprinting (Schütz et al., 2017; Ahlfeld et al., 2020). Alginic acid sodium salt (3 wt%; Sigma Aldrich, Taufkirchen, Germany) was dissolved into deionized water and sterilized by autoclaving at 120 °C for 20 min. Methylcellulose powder (9 wt%; Sigma Aldrich) and sea sand (30/50 wt%; sieved to particle size <0.25 mm; Carl Roth, Karlsruhe, Germany) were separately autoclaved at 120 °C for 20 min, before being manually stirred into the alginate solution with a spatula. The homogeneous paste was incubated for 2 h to allow the methylcellulose to swell. Hereafter, the hydrogel will be referred to as algMC + *x* wt% sand. After swelling, 100 µL per g hydrogel of either a bacterial suspension (7 × 10^7^ cells per gram bioink) or deionized water was added to the composite ink and mixed homogeneously.

Rheological properties were analyzed using a rotary rheometer (Rheotest RN 4.1, Medingen, Germany) with a plate-plate measurement setup and a plate distance of 0.5 mm. Viscosity and shear-thinning properties were determined by applying a constantly increasing shear rate from 0 to 100 s^-1^ for 300 s (n = 3). Shear recovery was characterized by applying a constant shear rate of 5 s^-1^ for 200 s, followed by a shear rate of 500 s^-1^ for 100 s. This procedure was repeated twice (n = 3).

### 2.3 Printability characterization by filament fusion and filament collapse test

Printability was characterized by the filament fusion and filament collapse test, described by Ribeiro et al. (2017). Briefly, the filament fusion test was performed by printing three layers of a meandering pattern with increasing strand spacing using the three-axial BioScaffolder plotter (BioScaffolder 3.1, GeSiM, Radeberg, Germany). The hydrogel was extruded through a nozzle with an inner diameter of 840 µm (Globaco, Rödermark, Germany). After plotting, the structures were imaged with a stereo light microscope (Leica M205 C with DFC295 camera, Wetzlar, Germany) and analyzed with the image software Fiji (ImageJ 1.53f51, Schindelin et al., 2012). Filament segment length and filament thickness were measured and the quotient of these measurements was evaluated as a function of the corresponding filament spacing. The measurements were performed with a sample size of n = 5.

The filament collapse test was performed by printing a single filament on a platform with pillars with the bioprinter BioScaffolder 3.1, with the gap distance increasing with every pillar (1, 2, 4, 8, 16 mm). Immediately after printing, a photo was taken and analyzed with the software Fiji. For the analysis, the angle of filament deflection was measured and plotted against the corresponding gap distance. The measurements were performed in triplicates.

### 2.4 Viability assessment and quantification of embedded cyanobacteria in the printed hydrogel scaffolds

The viability of PCC 7002 in bioprinted scaffolds was analyzed by fluorescence microscopy using the Keyence BZ-X700 (Keyence, Osaka, Japan). Living cells were determined by imaging the autofluorescence of chlorophyll (red). Dead cells were stained using the nucleic acid dye SYTOX Green (Invitrogen, Thermo Fisher Scientific, USA) (Sato et al., 2004; Schulze et al., 2011). First, scaffolds (10 mm × 10 mm, h = 3 mm) were printed with an air pressure of 200 kPa and a plotting speed of 8 mm/s, a layer-to-layer orientation of 90 °C and a layer thickness of 0.56 mm with a 840 µm needle and a strand distance of 1.45 mm. These were ionically crosslinked with 100 mM CaCl_2_ solution for 10 min and further cultured in 12-well plates (Corning, New York, USA) with 2 mL medium per well and scaffold. After certain cultivation time points, each scaffold was incubated in 2 mL deionized water containing 5 mM SYTOX Green for 15 min in the dark. Viability as a quotient of living cells to the sum of imaged cells was calculated using the image software Fiji.

To analyze the growth of the cyanobacteria in the bioprinted scaffolds, the chlorophyll content was determined. Bioprinted scaffolds were collected after 1, 3, 7, 10, and 14 days of cultivation and stored at −80 °C until further analysis. After thawing, 3 mL of 100 mM sodium citrate solution was added per scaffold to dissolve the constructs overnight at 4 °C. After centrifugation of the dissolved samples at 12,000 rpm for 15 min, the supernatant was removed. The cyanobacterial pellet was resuspended in 250 µL dimethyl sulfoxide (DMSO, Sigma) and transferred to a Precelllys tube (Peqlab, Erlangen, Germany), which was frozen at −80 °C for 20 min. After thawing, three ceramic Precelllys beads (Peqlab) and 1 mL DMSO were added to the tube. The tube was transferred to the cell homogenizer (Precelllys 24 system, Peqlab), where it was shaken three times for 30 s at 5,000 rpm. Into a transparent 96-well plate, 200 µL of the lysate was added per well and the optical density was measured at 435 nm with a microplate reader (Infinite M200 pro, Tecan, Switzerland). Measurements were performed in triplicates.

### 2.5 Analysis of biomineralization

#### 2.5.1 Biomineralization assay

The mineralization medium was prepared as described by Heveran et al. (2020). Briefly, an A+ medium without NaCl was prepared, and 100 mM NaHCO_3_ was added, followed by adjusting the pH to 7.6, after which 100 mM of CaCl_2_ was slowly added to the medium, which was finally sterile-filtered through a 0.2 µm cellulose acetate filter (Corning, New York, USA). Liquid culture biomineralization was performed by incubating 10 vol% of the preculture into the mineralization medium so that the initial optical density (OD) at 750 nm was 0.3. The suspension was cultured in the light incubator Infors Multitron for 24 h at 24 °C, 200 rpm, and 180 μmol m^-2^ s^-1^ illumination. For the biomineralization experiments with immobilized PCC 7002 2 mL of mineralization medium was added to each 3D-printed scaffold. The scaffolds (10 mm × 10 mm, h = 10 mm) were printed with an air pressure of 200 kPa and a plotting speed of 8 mm/s, a layer-to-layer orientation of 90 °C and a layer thickness of 0.56 mm with a 840 µm needle and a strand distance of 1.45 mm. After ionic crosslinking with 100 mM CaCl_2_ solution for 10 min, the scaffolds were cultured in the light incubator Infors Multitron at 24 °C, 200 rpm, and 180 μmol m^-2^ s^-1^ and removed from the medium for further analysis after 24 h, 7 days, and 14 days of cultivation.

#### 2.5.2 Scanning electron microscopy (SEM) and energy dispersive X-ray (EDX) analysis

SEM images were acquired using the Philips XL 30/ESEM (operated in SEM mode), at a voltage of 3 kV (spot size 3). Before imaging, liquid samples were dropped onto a 0.2 µm cellulose acetate filter and washed twice with deionized water. After fixation with 3.7% formaldehyde for 30 min, samples were dehydrated with increasing ethanol concentrations, air-dried under a fume hood, and sputter-coated with gold. 3D-printed scaffolds were directly air-dried and sputter-coated with gold. EDX measurements were conducted with samples prepared as described for SEM above.

#### 2.5.3 Mechanical properties

A uniaxial compressive test was performed to analyze the mechanical properties of fresh and dried 3D printed scaffolds after mineralization using a universal testing machine (Z010 with a 100 N and 10 kN load cell; Zwick, Germany). The scaffolds were fabricated as described above in 2.5.1. Fresh scaffolds were tested directly after removal from the mineralization medium, dried scaffolds were air-dried for 14 days at room temperature until weight equilibrium prior to the measurements. The uniaxial compressive test was performed with a speed of 0.2% l_0_ per min and the compressive strength was analyzed.

### 2.6 Statistical analysis

Statistical analysis was performed using the software GraphPad Prism (GraphPad Software, Boston, USA). Grouped data were analyzed by using one-way Analysis of Variance (ANOVA) and Tukey’s method for multiple comparisons. In the graphical and numerical results, data are presented as mean ± standard deviation. When differences between data points were *p* < 0.05, they were considered statistically significant.

## 3 Results

### 3.1 Characterization of bioinks with incorporated sea sand particles

To evaluate the suitability of extrusion-based bioprinting as a manufacturing method for LBM, an ink consisting of 3 wt% alginate and 9 wt% methylcellulose (algMC) was chosen, as it is known for its good printability, biocompatibility, and is well-established in the field of bioprinting of both mammalian and non-mammalian cells (Kilian et al., 2017; Seidel et al., 2017; Ahlfeld et al., 2020). The goal was to modify the ink with a support material that would later increase the stability of the LBM and serve as nucleation sites for CaCO_3_ biomineralization. Sand was chosen as the support material because it is commonly used as fine aggregates in conventional concrete (Bos et al., 2016; Deutsches Institut für Normung, 2021).

**FIGURE 2:**
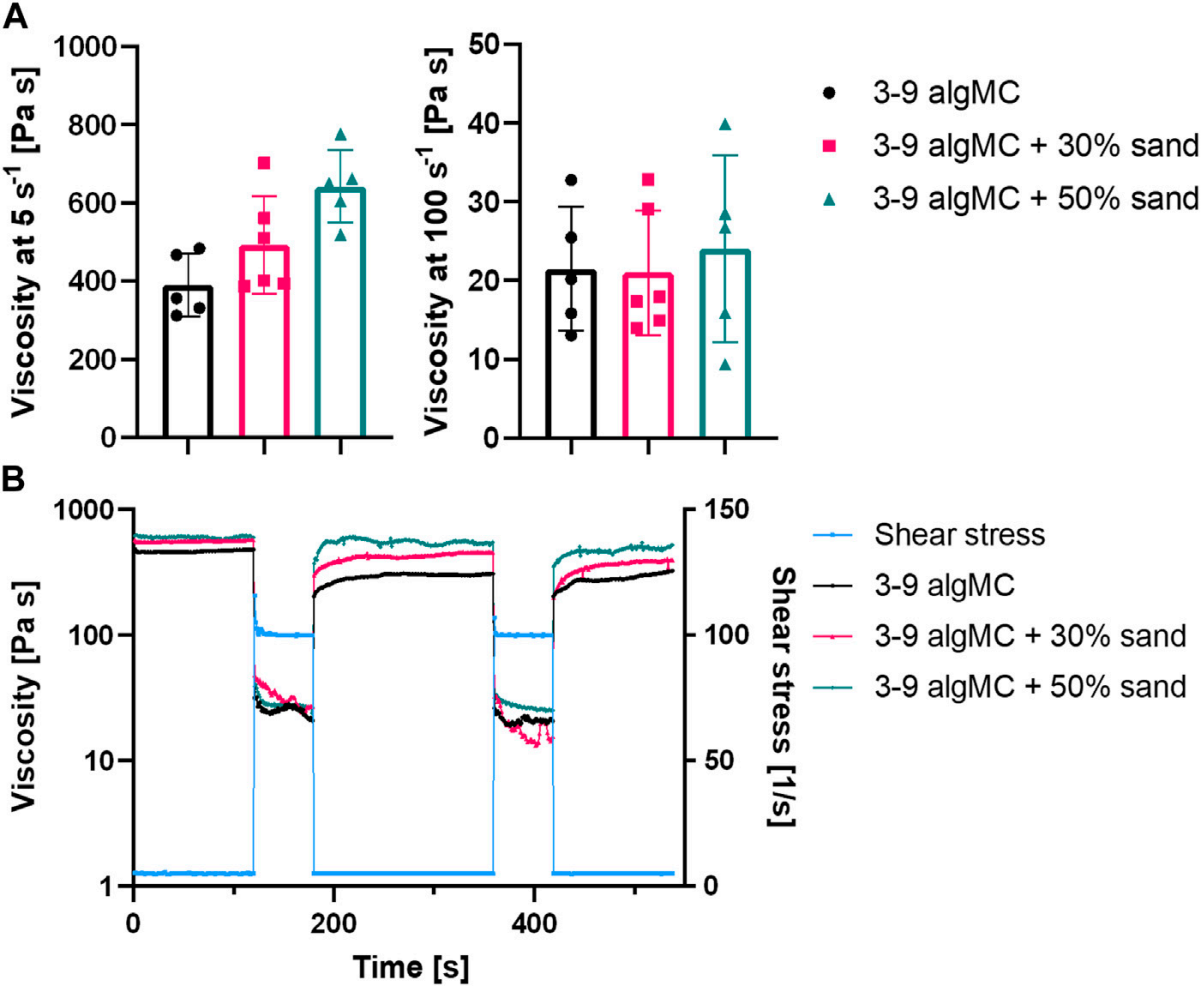
Rheological characterization of the 3 wt% alginate and 9 wt% methylcellulose (algMC) ink with the addition of 30 wt% and 50 wt% sand. **(A)** Viscosity of inks at low (5 s^-1^) and high (100 s^-1^) shear rates (mean ± SD, n ≥ 5); **(B)** Representative measurements of the shear recovery behavior.

The highest sand concentration tested in the algMC ink was 70 wt%. However, these composite hydrogels were very stiff and brittle, and therefore, not suitable for extrusion-based bioprinting. A homogenous paste was achieved with up to 50 wt% sand. To evaluate the suitability of the pastes for extrusion-based bioprinting, the viscosity and shear recovery were analyzed (Figure 2). The incorporation of sand did not affect the shear-thinning behavior of the paste, which is an essential property for printability in extrusion-based processes. At a low shear rate of 5 s^-1^, the viscosity increased with higher sand concentrations, from 390 ± 80 Pa s in algMC to 493 ± 125 Pa s with 30 wt% sand, and to 642 ± 94 Pa s with 50 wt% sand. At a high shear rate of 100 s^-1^, the viscosity with and without sand was approximately the same at 21 ± 8 Pa s, 21 ± 7 Pa s, and 22 ± 14 Pa s, respectively.

Furthermore, the shear recovery was analyzed to investigate the recovery potential of the algMC biomaterial inks with increasing sand concentration (Kesti et al., 2016; Paxton et al., 2017). A low (5 s^-1^) and high shear rate (100 s^-1^) were alternately applied, and viscosity was measured. Figure 2B shows that recovery in viscosity was observed for all hydrogels. However, after the first loading at high shear rates, the viscosity did not recover completely to the initial state at low shear rates. After further loading at high shear rates, it recovered to the state after the first high shear loading. This indicates the initial irreversible alteration of the polymeric structures (Ahlfeld et al., 2018).

**FIGURE 3:**
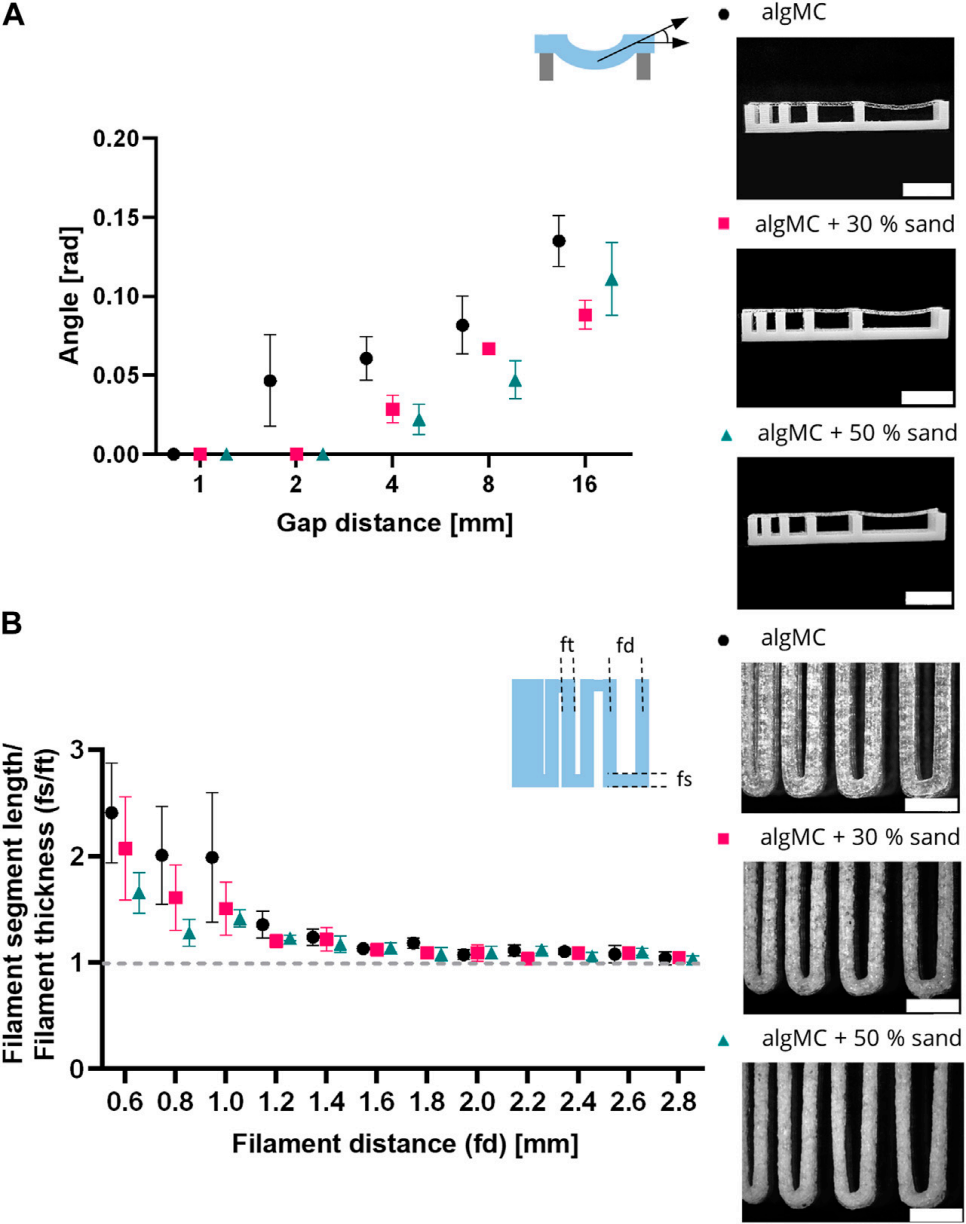
Printability characterization of the 3 wt% alginate and 9 wt% methylcellulose (algMC) ink with the addition of 30 wt% and 50 wt% sand, respectively. **(A)** Filament collapse test (mean ± SD, n = 3). Scale bar = 10 mm. **(B)** Filament fusion test (mean ± SD, n = 5). Scale bar = 4 mm.

To evaluate the shape fidelity of printed filaments, Ribeiro et al. proposed a filament collapse and filament fusion test (Ribeiro et al., 2017). Briefly, in the filament collapse test, a strand was printed on a bridge structure with increasing distance between the gaps (Figure 3A). The angle of the collapsing strand was measured using image analysis and plotted against the corresponding gap distance. The bending angle of the filament decreases with increasing sand content. At the maximum tested gap distance of 16 mm, the angle decreases from 0.135 ± 0.016 rad in algMC to 0.088 ± 0.016 rad with 30 wt% sand to 0.111 ± 0.023 rad with 50 wt% sand. The higher viscosity at low shear rates, as shown above in Figure 2A, makes the material stiffer after printing with increasing sand concentrations.

In the filament fusion test, three strands were printed on top of each other in a meandering pattern with increasing strand distances. For analysis, filament segment length and filament thickness were measured, and their ratio was plotted against the corresponding filament distance (Figure 3B). For all inks tested the ratio of the segment length to the thickness decreased with increasing strand distance. Above a filament distance of 2 mm, the ratio of filament segment length to filament thickness was about 1 for all inks tested, which is desirable. In general, the ratios are smaller for the inks with sand particles than for the algMC ink, indicating better shape fidelity.

### 3.2 Viability of bioprinted PCC 7002

**FIGURE 4:**
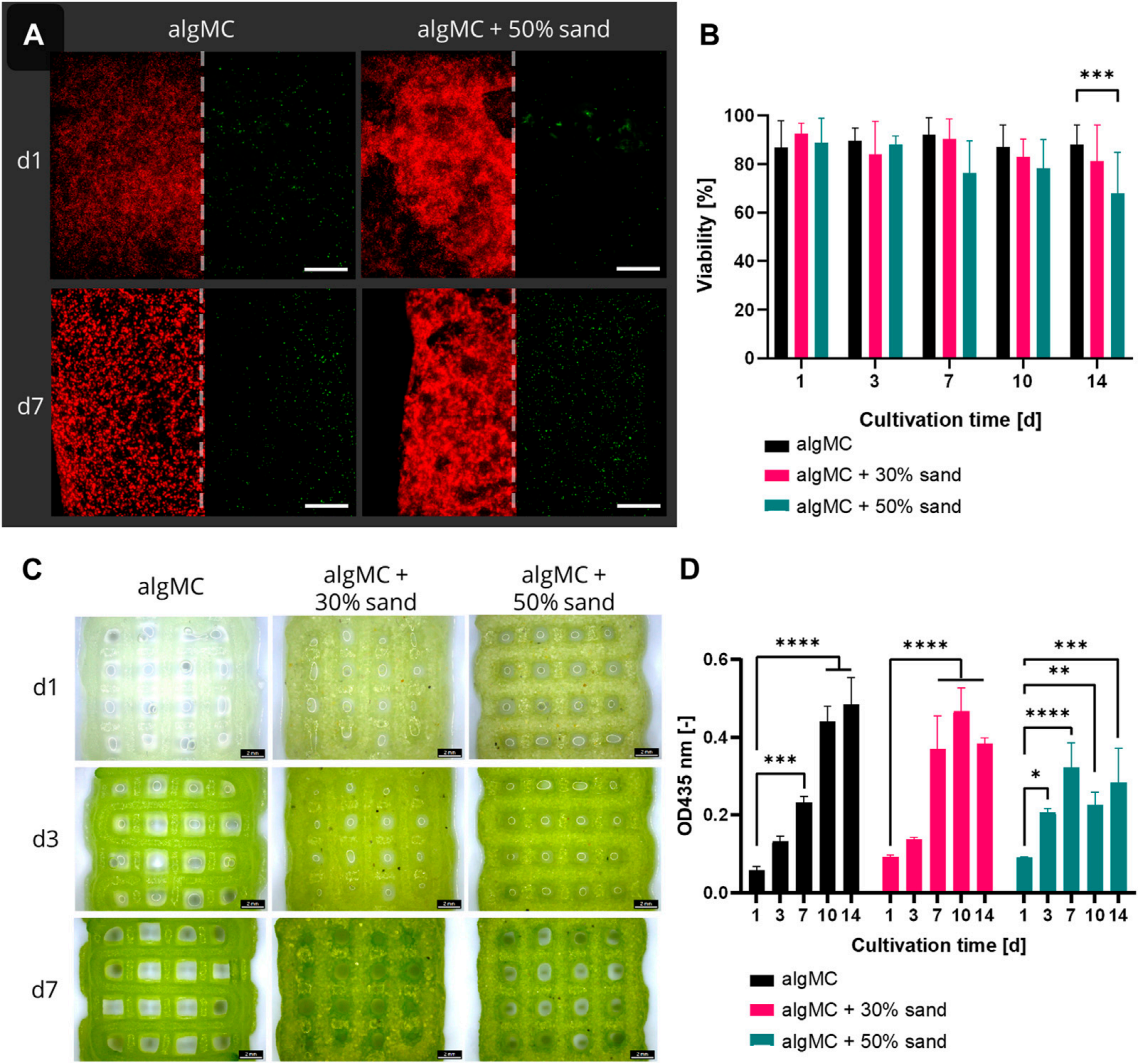
**(A)** Fluorescence microscope images of immobilized PCC 7002 in the algMC bioink with and without the addition of 50 wt% sand. Living cells show red autofluorescence (left), dead cells appear green (right). Scale bars = 200 µm. **(B)** Viability of PCC 7002 in the algMC bioink without, with 30 wt% and 50 wt% of sand, respectively, over a cultivation period of 14 days (data shows mean ± SD, n ≥ 5 of two individual experiments, ***p < 0.001). **(C)** Stereomicroscopic images of algMC scaffolds without with 30, and with 50 wt% sand after 1, 3, and 7 days of cultivation. Scale bar = 2 mm. **(D)** Measurement of the optical density (OD) at 435 nm (chlorophyll content) over a cultivation period of 14 days (data shows mean ± SD, n = 3, *p < 0.05, **p < 0.01, ***p < 0.001, ****p < 0.0001).

Since all ink formulations showed good printability and shape fidelity, *Synechococcus* sp. strain PCC 7002 (further referred to as PCC 7002) was added at a cell density of 7 × 10^7^ cells per gram bioink to analyze the effect of the hydrogel and sand particles on the cell viability during a 14-day cultivation period. The viability in the bioprinted constructs was determined by image analysis of live/dead stained fluorescence images taken after 1, 3, 7, 10, and 14 days of cultivation (Figure 4A). Additionally, stereomicroscopic images (Figure 4C) and the chlorophyll content (Figure 4D) of the scaffolds on each time point were analyzed. Overall, the viability was very high for all tested bioinks, being 88.7 ± 2.2% (algMC), 86.2 ± 4.9% (algMC +30 wt% sand), and 79.9 ± 8.7% (algMC +50 wt% sand). No significant difference in cell viability between the three bioinks could be detected until day 10 (Figure 4B). Only on day 14 the viability of PCC 7002 in the ink with 50 wt% sand (68.0 ± 16.7%) was significantly lower than in the ink without sand (88.0 ± 8.2%). As on day 1 the viability was high in all tested bioinks (86.8–92.6%), the incorporated sand particles did obviously not damage the cyanobacteria through increased shear stress during the printing process. The chlorophyll content (optical density (OD) at 435 nm) increased significantly for all tested bioinks within the first 7 days of cultivation (Figure 4D). By this time, the OD at 435 nm increased 4.1 ± 0.2-fold, 4.1 ± 0.8-fold, and 3.6 ± 0.6-fold compared to day 1 of cultivation in the algMC, algMC +30% sand, and algMC +50% sand bioinks, respectively. However, after 10 days of cultivation, cell outgrowth into the medium was observed in the samples with sand. This effect was stronger with increasing sand concentration and led to decreased chlorophyll measurements inside the scaffolds. After 14 days of cultivation, the OD at 435 nm was 0.48 ± 0.06, 0.38 ± 0.01, and 0.28 ± 0.07 in the algMC, algMC +30% sand, and algMC +50% sand bioinks, respectively.

### 3.3 Biomineralization in liquid culture and in bioprinted constructs

**FIGURE 5:**
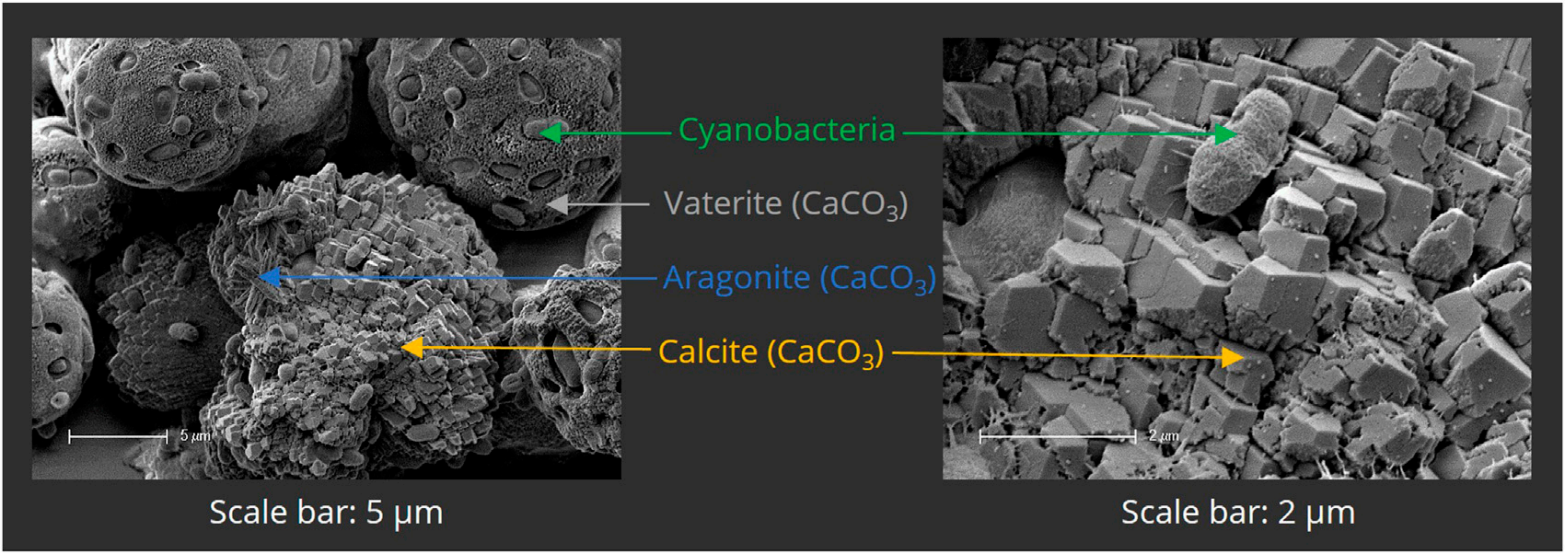
Scanning electron micrographs of mineralized PCC 7002 after 7 days of mineralization. Bacterial cells are surrounded by mineralized calcium carbonate (CaCO_3_), present in the different polymorphs vaterite, aragonite and calcite.

To ensure the calcifying ability of PCC 7002, a mineralization assay was first performed in liquid culture. Bacteria were cultured for 24 h in mineralization medium, containing 100 mM CaCl_2_ and 100 mM NaHCO_3_. The samples were then analyzed by SEM (Figure 5) and EDX (not shown). Calcium-containing minerals were observed adjacent to or incorporating the bacterial cells, indicating microbial calcification. CaCO_3_ was present in three different polymorphs—calcite, vaterite, and aragonite—that clearly can be distinguished by their morphological appearance by SEM as shown in Figure 5.

**FIGURE 6:**
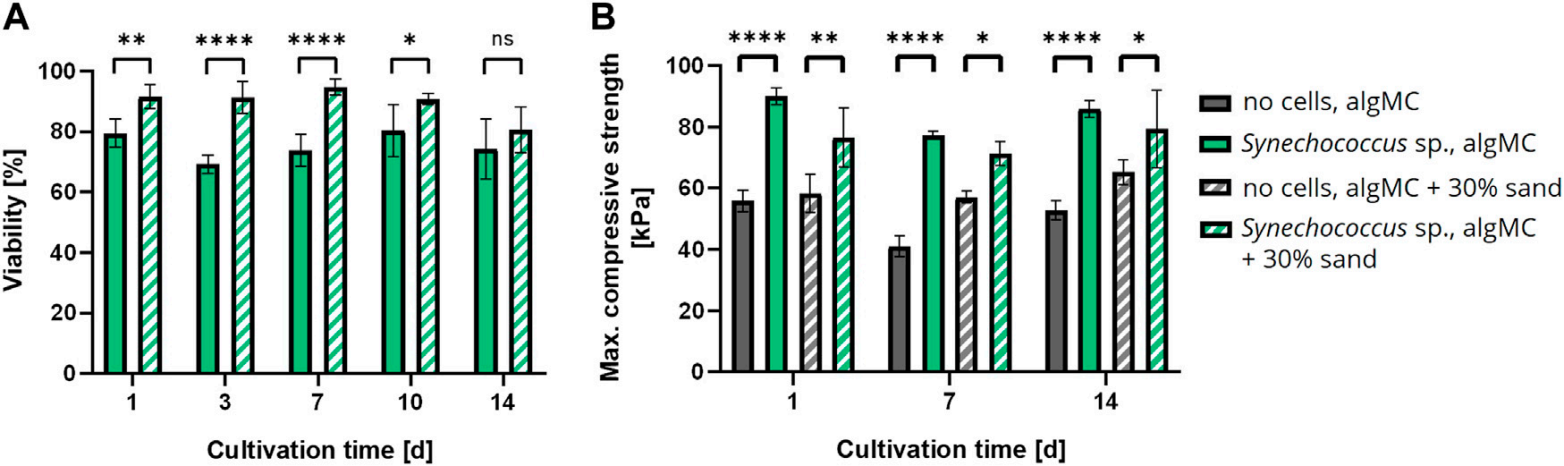
**(A)** Viability of bioprinted PCC 7002 in the algMC bioink without and with 30 wt% of sand over a cultivation period of 14 days (mean ± SD, n = 6, ****p < 0.0001, **p < 0.01, *p < 0.1). **(B)** Maximum compressive strength of wet scaffolds without cells (=abiotic control) and with PCC 7002 (=LBM) cultivated in mineralization medium for 1, 7, and 14 days (mean ± SD, n = 3, ****p < 0.0001, **p < 0.01, *p < 0.1).

Since biomineralization was observed in liquid culture, the next step was to induce biomineralization of the immobilized cells in the bioprinted constructs to create the LBM. Briefly, PCC 7002 was incorporated into the algMC bioink without and with 30 wt% of sand, at a cell density of 7 × 10^7^ cells per gram bioink. After printing, the scaffolds were crosslinked for 10 min with 100 mM CaCl_2_ and cultured in mineralization medium at 24 °C for up to 14 days. Cell viability was analyzed by fluorescence microscopy with image analysis and showed high viability in the LBM without and with 30 wt% of sand even after 14 days of cultivation in the mineralization medium, with a viability of 74.2 ± 9.8% and 80.6 ± 7.6% (n = 6), respectively (Figure 6A). A significantly higher viability in the scaffolds with 30 wt% sand was observed until day 10. However, after 14 days of cultivation, no significant difference regarding cell viability was measured between these bioinks.

In addition, the LBM and abiotic control (reference samples without cells) were mechanically analyzed by a uniaxial compression test. The samples were removed from the mineralization medium after 1, 7, and 14 days and analyzed in wet conditions. The maximum compressive strength of all tested samples did not change significantly over time. However, all LBM with PCC 7002 showed significantly higher maximum compressive strength than the abiotic control at all time points (Figure 6B). After 1 day of mineralization, the maximum compressive strength of the LBM was about 38% and about 24% higher than in the abiotic control without and with 30 wt% of sand, respectively.

**FIGURE 7:**
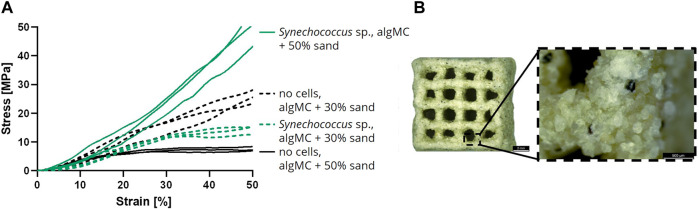
**(A)** Compressive stress over increasing strain of air-dried samples after the cultivation for 7 days in mineralization medium (n = 3). **(B)** Light microscope image of a dry mineralized sample with PCC 7002 and 50 wt% of sand. Scale bars represent 2 mm (left) and 500 µm (right).

In addition to the analysis of the LBM in wet condition, air-dried LBM and abiotic control samples were also examined for their mechanical properties. For this assay, samples were printed with 30 and 50 wt% of sand, respectively, with and without cells, cultured in mineralization medium for 7 days and air-dried until weight equilibrium (Figure 7B). A uniaxial compression test was performed with the dry LBM and the abiotic control (Figure 7A). When comparing the stress at 50% strain, the lowest values were measured for the abiotic control with 50 wt% sand (7.6 ± 0.8 MPa). The abiotic control with 30 wt% sand resulted in higher compressive strengths (25.7 ± 2.4 MPa). The LBM with PCC 7002 with 30 wt% sand had lower compressive strengths than the abiotic control (14.4 ± 1.5 MPa), but the LBM with 50 wt% sand yielded the highest compressive strengths measured (50.1 ± 6.3 MPa), 6.6 times higher than the respective control without cyanobacteria.

**FIGURE 8:**
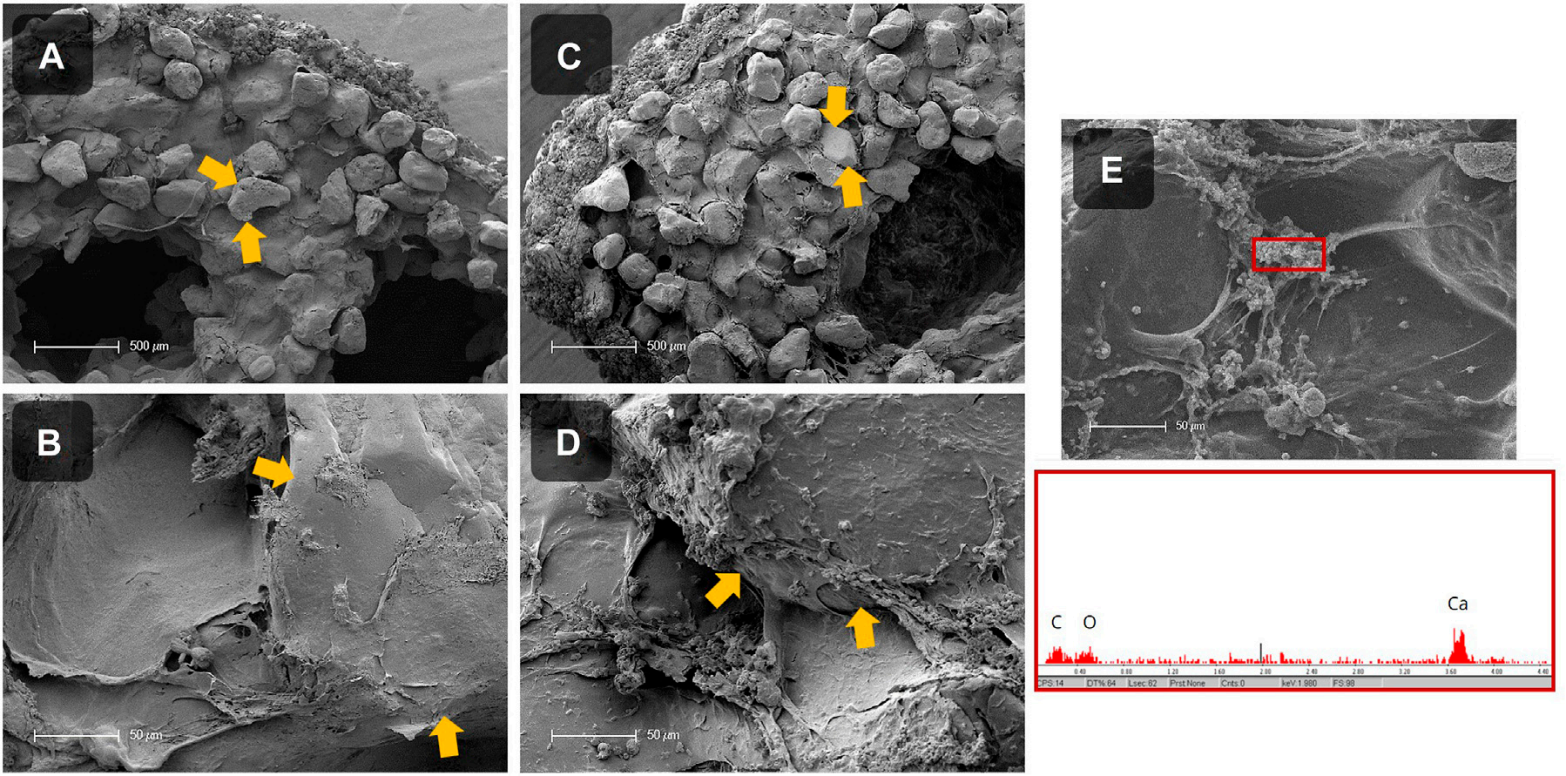
SEM images of the cross section of dried, mineralized scaffolds without cells (= abiotic control; **(A, B)**) and with PCC 7002 (= LBM; **(C–E)**) with 50 wt% of sand. Sand particles are marked with yellow arrows. EDX measurement of precipitates inside of the scaffold with PCC 7002, showing a calcium peak **(E)**. Scale bars **(A, C)** = 500 μm, scale bars **(B, D, E)** = 50 µm.

Besides the mechanical analysis, the mineralization inside the LBM was investigated by SEM and EDX measurements (Figure 8). Therefore, cross sections of LBM and abiotic control samples with 50 wt% sand were prepared, air-dried, sputtered with gold, and further analyzed. It was noticeable that the surfaces of both samples were covered with minerals. Inside the abiotic control, the alginate matrix connecting the sand particles looked smooth and almost no mineral precipitates were detected (Figures 8A, B). In contrast, many minerals were visible in the LBM (Figures 8C, D). Analysis of the mineral phases by EDX revealed the presence of a calcium peak (Figure 8E), indicating the formation of calcium carbonate.

## 4 Discussion

Cyanobacteria have not only contributed greatly to the emergence of aerobic life forms in the past but are still of great importance today, especially for the capture and storage of CO_2_ from the atmosphere (Falkowski, 1994; Whitman et al., 1998; Tetsch, 2016). Their ability to mineralize CaCO_3_ and simultaneously sequester CO_2_ is a promising functionality that has been exploited within the concept of Living Building Materials (LBM) in this study to create environmentally friendly building materials. 3D bioprinting was investigated as a manufacturing method to produce LBM, since recent studies analyzing 3D bioprinting of bacteria, such as *Escherichia coli* (Lehner et al., 2017), *Bacillus subtilis* (Huang et al., 2019), or *Acetobacter xylinum* (Schaffner et al., 2017)*,* demonstrated good cell viability and functionality over days up to several weeks. A blend of alginate and methylcellulose (algMC) was chosen as the hydrogel matrix of the LBM because it is a well-established bioink for extrusion-based bioprinting that exhibits high biocompatibility with various mammalian and non-mammalian cell types (Lode et al., 2015; Ahlfeld et al., 2017; Schütz et al., 2017; Dani et al., 2022). Alignate as a natural polymer has been used as an additive to mortar in several studies, which resulted in an increased compressive and tensile strength (Susilorini et al., 2014; Mohesson and Abbas, 2020) and enhances the building materials flame-, fire-, and heat-resistance significantly (DeBrouse, 2012). Further studies analyzed methylcellulose as an admixture to conventional concrete (Fu and Chung, 1998; Ahmed et al., 2021; Liu et al., 2021) and clay-based cementitious materials (Chen et al., 2020). It resulted in increased tensile strength, flexural properties, adhesion to steel reinforced concrete (Fu and Chung, 1998), enhanced plastic viscosity, and optimized rheological parameters for 3D printing applications of conventional concrete materials (Douba and Kawashima, 2021; Liu et al., 2021). Sea sand was added as a structural support component that should be consolidated by biomineralized CaCO_3_, as sand is a cheap commercial and common additive in construction materials such as concrete (Bos et al., 2016; Deutsches Institut für Normung, 2021). Rheological analyses revealed that up to 50 wt% sand can be added to the algMC ink, not affecting the shear-thinning behavior as an essential property for printability in extrusion-based 3D printing. At low shear rates (5 s^-1^) the viscosity increased with increasing sand concentration. However, at high shear rates (100 s^-1^) sand concentration did not affect the viscosity. These results are consistent with those of a study from Spangenberg et al. in which up to 50 wt% magnetite microparticles (mean diameter 25–30 µm) were incorporated into an algMC bioink while maintaining shear-thinning properties (Spangenberg et al., 2021). However, 3D printing of the bioinks with more than 25 wt% magnetite resulted in lower shape fidelity of the printed structures. In contrast, all hydrogels tested in this study exhibited good shear recovery properties and a high shape fidelity. Sand concentrations at and above 70 wt% resulted in stiff and brittle gels that were not printable. To make inks with higher solid particle concentrations printable, further adjustments would be required, such as varying the methylcellulose concentration and the particle size distribution. For further improvement of the environmental sustainability of the ink, alternative support materials to sea sand could be used, such as fly ash, cement kiln dust, silica fume, limestone, microfibers, or slag (Bos et al., 2016; Irfan et al., 2019; Kaufhold et al., 2019; Bhattacherjee et al., 2021; Booth and Jankovic, 2022).

Subsequently, algMC-based inks were tested with the cyanobacterium *Synechococcus* sp. strain PCC 7002 (further referred to as PCC 7002). As the viability after the extrusion printing was still very high (86.8–92.6%) for all tested inks, PCC 7002 seem to tolerate the printing pressure during bioprinting. Even the addition of 50 wt% sand particles, which increase the shear forces during printing, did not reduce the viability of the cells. The significant increase in the chlorophyll content of the immobilized cells during the first 7 days of cultivation confirms the functionality of PCC 7002 regarding cell proliferation and photosynthetic activity. During prolonged cultivation, the cells increasingly grew out of the scaffolds with incorporated sand particles into the surrounding cell culture medium. This effect increased with higher sand concentrations. Since the methycellulose is not crosslinked and remains soluble, it is washed out of the scaffolds into the medium with increasing cultivation time, leading to the formation of micropores (Ahlfeld et al., 2020), from which the cells can grow out of the scaffold. Since the sand particles also do not chemically react with the algMC matrix, this might lead to interstitial spaces and additional pores between the sand and the alginate network, increasing cell outgrowth. This effect could have led to the apparent decrease in viability after 14 days of cultivation between the bioink without and with 50 wt% of sand. To prevent the outgrowth, a thin dip coating with, for example, pure alginate could be used, which however might lead to a reduced exchange between the bioink strands and the surrounding medium. Additionally, cell viability of bioprinted PCC 7002 was also analyzed in mineralization medium, where it also was very high throughout the 14-day cultivation period (74.2–80.6%), confirming the stability of PCC 7002 against high salinities (Ludwig and Bryant, 2012). Since the mineralization process of cyanobacteria is fast and takes place within a few days or even hours, the viability tested in this study is considered high enough for the fabrication of the LBM (Heveran et al., 2020; Sidhu et al., 2022; Yang et al., 2022).

Through analysis of biomineralization in liquid culture, three different polymorphs were observed, namely, calcite, vaterite, and aragonite. It is known that different phases of CaCO_3_ are formed during bacterial precipitation of calcium carbonate (Rodriguez-Navarro et al., 2007; Rodriguez-Navarro et al., 2012; Dhami et al., 2013a; Hirsch et al., 2023), calcite and aragonite being the most abundant and stable ones (Leeuw and Parker, 1998). Interestingly, most of the natural occurrences of vaterite are associated with biogenic processes (Lippmann, 1973; Mann, 2005). Through scanning electron microscopy (SEM), structures surrounding the cells and interconnecting with the mineral phases were observed. The substances might be extracellular polymeric substances (EPS), which are known to be produced by a wide range of cyanobacteria (Nicolaus et al., 1999; Philippis et al., 2001; Pereira et al., 2009) and can largely influence the polymorphism of the precipitated CaCO_3_ (Kawaguchi and Decho, 2002). Yu et al. reported the importance of the EPS produced by PCC 7002 for the sequestration of caesium ions due to several functional groups, such as amino-, hydroxyl-, and phosphate groups (Yu et al., 2020). Swanner et al. observed similar morphological structures and determined that they might be hydroxamate- and catechol-type siderophores, produced due to iron deficiency (Swanner et al., 2015). Machado et al. observed EPS-like fibrin structures in PCC 7002 cultures, as the bacteria were exposed to nano- and micro-polyethylene particles (Machado et al., 2020). Chekroun et al. found that spherical vaterite is mainly present with live bacteria during mineralization (Chekroun et al., 2004). Vaterite production is strongly promoted by EPS (Naka and Chujo, 2001), especially by those presenting amino (Braissant et al., 2003; Ichikawa et al., 2003) or phosphate groups (Dupont et al., 1997; Sawada, 1997). These negatively charged groups could have attracted calcium ions and enhanced mineral formation in this study. However, further analysis must be done to identify and characterize the EPS-like structures.

Sidhu et al. analyzed the three-dimensional consolidation of a sand column through biomineralization by the cyanobacterium *Synechocystis pevalekii* and detected CaCO_3_ precipitation plugging the pores between the sand particles. However, the consolidation was inhomogeneous because of the lack of light and aeration in parts of the column (Sidhu et al., 2022). 3D bioprinting could prevent these inhibitions through the incorporation of targeted macropores, i.e., the voids between the extruded strands. Therefore, after successful mineralization in liquid culture, the next step was to induce biomineralization in the bioprinted scaffolds to create the LBM. Mechanical testing, SEM imaging, and EDX measurements were performed to characterize the mineralization process and the resulting material composites. The uniaxial compression test of wet scaffolds showed that the maximum compressive strength of the LBM with PCC 7002 was higher than that of the abiotic control group without cells at each time point tested, namely, after 1, 7, and 14 days of cultivation in the mineralization medium. This increase indicates the promotion of mineralization by the incorporated bacteria. However, no further increase in compressive strength was measurable over time. This suggests that the mineralization process was already finished after 24 h, due to a potential equilibrium between calcium ions solidified in CaCO_3_ and calcium ions crosslinking the alginate matrix, by forming egg-box junctions with the polyguluronate units of the alginate polysaccharide chains (Merakchi et al., 2019). To overcome this limitation, biomineralization could be designed as a multiple-step process in which new mineralization medium is added every 24 h, or as a continuous process in which the mineralization medium constantly flows through the porous scaffolds.

When testing the mechanical strength of air-dried samples, the abiotic control showed lower compressive strength with increasing sand concentration. This initially astonishing observation could be explained by the fact that the lower gel-to-sand ratio in the biomaterial blend with 50 wt% sand likely resulted in thinner (i.e., less stable) gel bridges between the sand particles, which break at lower stress than in the blend with 30 wt% sand. However, when PCC 7002 cells were added, the dependence reversed. This indicates that the bacteria-induced CaCO_3_ mineralization was sufficient to consolidate the samples with a higher content of solid filler particles and therefore, a lower particle-to-particle distance that could be overcome by the biogenic mineral precipitates. Heveran et al. also confirmed the improvement of the mechanical strength of structures with PCC 7002, resulting in a 15% higher fracture energy for the LBM than for the abiotic control (Heveran et al., 2020). Several other studies have revealed the importance of microorganisms in the mineralization process (Rodriguez-Navarro et al., 2007; Bundeleva et al., 2014). However, the microorganisms themselves may already be contributing to this effect (Skevi et al., 2021), as Zhang et al. observed a similar effect on fracture toughness when they compared the addition of a mineralizing strain and a wild-type control into a cementitious matrix (Zhang et al., 2019). Whether PCC 7002 actively or passively affected the mineralization processes and mechanical properties of the LBM in this study, is not yet clear. This could be further analyzed with a dead or inactivated cell control group.

SEM images showed that calcium carbonate precipitated in the LBM as well as in the abiotic control because the mineralization medium was supersaturated with calcium chloride and sodium bicarbonate. Since the outside scaffold serves as a nucleation point for the mineral production, a homogeneous mineral layer was observed across the entire surface. However, morphological differences between the biotic and abiotic samples inside the strands were evident in the scaffold cross-sections. The matrix between the sand particles in the abiotic control was smooth, and only few mineral particles were visible. In contrast, in the LBM CaCO_3_ was found surrounding the cells and aligning along the alginate matrix between the sand grains, EDX measurements confirmed CaCO_3_ precipitation. Obst et al. showed that CaCO_3_ precipitates at the outer surface of the microorganism, interconnecting with the cyanobacterial EPS, possibly leading to changes in the mechanical properties of LBM compared to abiotic control samples (Obst et al., 2009).

In conclusion, the biomineralizing strain PCC 7002 was successfully incorporated into an alginate-based bioink containing up to 50 wt% sand as support material and macroporous scaffolds were fabricated by extrusion-based 3D bioprinting. The cells showed high viability in the immobilized state over a cultivation period of 14 days and remained functional regarding their MICP capability. Mineralized LBM were successfully fabricated and showed increased compressive strength when living cells were present. This indicates the consolidation of the sand particles through mineralized CaCO_3_, which presence was confirmed by microstructural analyses of the LBM.

## Data Availability

The raw data supporting the conclusion of this article will be made available by the authors, without undue reservation.
